# Implications of wait times for sentinel node biopsy on melanoma disease progression, micrometastatic tumour burden and survival outcomes in the modern treatment era

**DOI:** 10.1038/s41416-026-03497-9

**Published:** 2026-06-16

**Authors:** Sofia O. Breeze, Martin J. Heaton, Andrew P. Snelling, Jennifer J. Garioch, Jenny P. Nobes, Marc D. Moncrieff

**Affiliations:** 1 Norwich Medical School, University of East Anglia, Norwich, UK; 2 Department of Plastic & Reconstructive Surgery, Norfolk and Norwich University Hospitals NHS Foundation Trust, Norwich, UK; 3 Department of Dermatology, Norfolk and Norwich University Hospitals NHS Foundation Trust, Norwich, UK; 4 Department of Oncology, Norfolk and Norwich University Hospitals NHS Foundation Trust, Norwich, UK

**Keywords:** Surgical oncology, Outcomes research, Melanoma, Melanoma

## Abstract

**Background:**

Sentinel node biopsy (SNB) predicts long-term melanoma outcomes and advises referrals for adjuvant systemic therapy (AST). Concerns regarding the impact of treatment delays on clinical outcomes remain.

**Methods:**

A UK retrospective cohort study at a supra-regional centre was performed, including 1625 patients listed for SNB between 2010-23. Three parameters were investigated: disease progression, micrometastatic burden, and disease-specific survival. Time-to-SNB was stratified as early (≤90days) or late (>90days), with a subgroup analysis accounting for AST use (2017-23). Sensitivity analyses calculated a wait-time threshold beyond which worsens survival outcomes.

**Results:**

Median time-to-SNB was 68 days (IQR = 51–95) and follow-up 75 months (IQR = 35–115). Late surgery correlated with disease progression before surgery (0.34% versus 1.54%; OR = 4.31; *p* = 0.022); larger micrometastases (1.40 mm vs. 2.95 mm; *p* = 0.003), and increased high-risk micrometastatic deposits >1 mm (55.2% vs. 69.1%; OR = 2.04; *p* = 0.014). For patients post-2016, longer wait-times correlated with poorer DSS (HR = 1.01 (1.00–1.02); *p* = 0.011 for diagnosis-to-SNB; and HR = 1.01 (1.00–1.02); *p* = 0.016 for primary excision-to-SNB). Sensitivity analyses calculated ≤68-days from diagnosis (HR = 3.17 (1.19–8.46); *p* = 0.021) and ≤82-days from excision (HR = 2.94 (1.10–7.87); *p* = 0.032) as optimal treatment thresholds.

**Conclusion:**

Surgical delays correlated with harm: disease progression, increased tumour burden and worse survival. SNB should occur within 68 days from diagnosis and/or 82 days from excision biopsy.

## Introduction

For many countries, melanoma is a growing health concern. Incidence rates have climbed by 31% in the last 10 years in the UK [1], meaning that skin cancer centres are struggling to compete with increasing demands, particularly since specialist cancer services are largely centralised. Sentinel node biopsy (SNB) is an important staging procedure that is the current standard care internationally for American Joint Committee on Cancer (AJCC) [2] pT1b-pT4b primary cutaneous melanomas, and guides referrals for further treatment. The National Comprehensive Cancer Network (NCCN) and Surgical Society of Oncology proposed a 90-day threshold within which wide-local excision and SNB should take place from the time of melanoma diagnosis [3, 4], however, the evidence-base for this decision is limited. Anecdotally, patients are routinely waiting longer than 90 days for their surgery across the UK. Currently, the conflicting evidence fails to provide definitive evidence for harm with such delays, thus making it difficult for clinicians to argue for the allocation of resources to tackle excessive delay in current SNB waiting lists.

SNB is indicated in approximately 45% of invasive cutaneous melanoma cases, currently affecting around 7900 individuals annually in the UK [1, 5]. It is not only a key staging procedure but also may confer therapeutic benefit, as suggested by MSLT-1 data, particularly in selected patients [6–8]. Those with low micrometastatic tumour burden (deposit diameter <0.3 mm) can be managed similarly to SNB negative patients of the same AJCC T-stage, thereby avoiding adjuvant systemic therapy (AST) and radiological surveillance [5]. Conversely, patients with higher micrometastatic tumour burden (deposit diameter >1 mm) should be referred for consideration of AST, in line with current NICE and NCCN guidelines [5, 9]. Accordingly, we hypothesise that patients experiencing delays in the SNB pathway may experience harm in three key ways. Patients’ disease may progress on the waiting list requiring more extensive treatment; increased micrometastatic tumour burden leading to more frequent referrals for AST; and poorer survival outcomes. No studies have yet assessed these important endpoints in conjunction, or determined the time point at which delays in the pathway increase the risk of harm.

This study aims to quantify the impact of surgical timing on key clinical outcomes in melanoma, assess the risk of harm from delayed surgery, and define a time threshold for SNB to inform future guidelines.

## Methods

This research was approved by the UK Health Research Authority (IRAS ID: 332188) and the protocol was reviewed by the East of England (Essex) Research Ethics Committee (Ref: 23/EE/0283). In this retrospective cohort study, data was collated on patients from our prospective institutional melanoma database with primary cutaneous melanoma who were listed for a SNB alongside wide local excision between 1^st^ January 2010- 31^st^ December 2023 at our tertiary cancer centre in the East of England. Adult patients (≥18 years) with pT1b-pT4b (AJCC 8^th^ Edition) primary cutaneous melanomas without synchronous regional and/or distant disease at baseline were included in this study [5, 9]. Those with mucosal, oral, genital or rare melanoma subtypes (e.g., melanocytic lesions of unknown malignant potential, atypical spitz etc.), microsatellites at diagnosis, co-existent primary melanomas and known metastatic melanoma were excluded from the dataset.

Standard patient demographic data and primary tumour characteristics were recorded (Table 1). The primary study exposure was the time (days) between the date of diagnosis (date of the diagnostic pathology report) to the date of SNB (time-to-SNB). The time (days) from date of primary diagnostic excision to reporting (reporting interval) and to SNB (diagnostic excision biopsy-to-SNB) were also noted. The 90-day cut-point was used to stratify patients into two-time cohorts: early (≤90 days) or late (>90 days). The planned or actual date of surgery was the time of primary intervention. Patients were treated as per national melanoma guidelines, and as such no routine imaging is indicated at baseline for this stage of melanoma [5]. Any disease progression and/or recurrence was identified during routine follow-up and/or by imaging with histological confirmation when clinically indicated after diagnosis.

**Table 1:** Cross-table comparing patient demographic and primary tumour characteristics for patients at the time of diagnosis with an early (>90 days) or late (≤90 days) SNB for the entire cohort (*n* = 1625).

Variable	*N*	Early SNB (≤90 days) (n = 1170)		Late SNB (>90 days) (n = 455)		Test statistic	
							P-value
**Age (yrs), median (IQR)**	1625	64	(52-71)	65	(55-72)	F_1,1623_ = 1.94	0.163^a^
**Breslow thickness (mm), median (IQR)**	1622	1.8	(1.2-3.0)	1.9	(1.2-3.4)	F_1,1620_ = 2.65	0.104^a^
**Reporting interval (days), median (IQR)**	1625	9	(6-14)	12	(8-16)	**F**_**1,1623**_ = **38.64**	**<0.001**^**a**^
**Sex: Male**	1625	610	(52.1)	233	(51.2)	X^2^_1_ = 0.11	0.737^b^
**Referral location: Peripheral hospital**	1625	570	(48.7)	303	(66.6)	**X**^**2**^_**1**_ = **42.10**	**<0.001**^**b**^
**Site**	1625					X^2^_3_ = 0.52	0.913^b^
Head and neck		176	(15.0)	70	(15.4)		
Lower extremity		295	(25.2)	108	(23.7)		
Torso		453	(38.7)	176	(38.7)		
Upper extremity		246	(21.0)	101	(22.2)		
**Year: Post-2016**	1625	384	(32.8)	346	(76.0)	**X**^**2**^_**1**_ = **247.37**	**<0.001**^**b**^
**Subtype**	1625					X^2^_4_ = 2.65	0.617^b^
Superficial spreading		830	(70.9)	313	(68.6)		
Nodular		203	(17.4)	89	(19.6)		
Lentigo		52	(4.4)	20	(4.4)		
Acral lentiginous		25	(2.1)	6	(1.3)		
Other		60	(5.1)	27	(5.9)		
**Lymphovascular invasion: Yes**	1598	77	(6.6)	20	(4.4)	X^2^_1_ = 2.69	0.101^b^
**Ulceration: Yes**	1621	298	(25.5)	121	(26.6)	X^2^_1_ = 0.21	0.645^b^
**T-stage**^**b**^	1620					X^2^_4_ = 9.25	0.055^b^
IA		35	(3.0)	10	(2.2)		
IB		580	(49.6)	208	(45.7)		
IIA		266	(22.7)	114	(25.1)		
IIB		192	(16.4)	67	(14.7)		
IIC		93	(7.9)	55	(12.1)		
**SNB Performed : Yes**	1625	1,103	(73.1)	407	(27.0)	**X**^**2**^_**1**_ = **11.59**	**<0.001**^**b**^

Values are *n* (%) unless otherwise indicated; *N*; number of non-missing values; SNB, sentinel lymph node biopsy; IQR, interquartile range; N, number of patients.

^a^Kruskal-Wallis 2 Pearson.

^b^American Joint Committee on Cancer (AJCC) Classification.

Statistically significant results in bold.

The study design assessed three key outcomes: disease progression, micrometastatic disease burden and disease-specific survival. Several patients who were listed for SNB ultimately did not have the procedure. The underlying cause for this outcome was sought, specifically whether it was related to ‘progression’, defined as clinical and/or radiological metastatic disease after primary diagnosis and prior to or at the time of lymphoscintigraphy. Micrometastatic tumour burden was recorded using important phenotypic characteristics: nodal positivity, the number of affected nodes (AJCC 8^th^ edition N-stage), extracapsular spread (ECS) and maximum sentinel node deposit diameter (mm). High-risk deposits were described as >1 mm in maximum deposit diameter [10].

Disease-specific survival (DSS) was the primary survival outcome, and recurrence-free survival (RFS) and distant-metastasis free survival (DMFS) were also recorded. These were measured from the time (months) of primary melanoma treatment (date-of-SNB) to the matching endpoint, last follow-up by the skin cancer MDT, or censor date. The date of melanoma-related death was the outcome for DSS. RFS was recorded as the time taken for a local, regional or distant recurrence (the earliest date of pathology or radiology report) recorded as the most severe type at the time, and distant recurrences for DMFS. Patients received routine follow-up. To limit drop-out bias, in cases of incomplete follow-up the date of last review was noted, and outcomes were censored up to and including 31^st^ December 2023.

### Statistical analysis

Statistical analyses were performed using Jamovi (version 2.5, Sydney, Australia) [11] using R project computing language (version 4.3, open sources https://cram.r-project.org) [12]. The data were stratified by whether SNB was performed early (≤90 days) or late (>90 days). A pre-planned subgroup sensitivity analysis for patients treated post-2016, periods where systemic therapy became the standard of care at our centre. Using time-to-SNB, both as a continuous and stratified variable, multivariable logistic regression was utilised to adjust for known prognostic factors. Kaplan-Meier log-rank method [13] and Cox’s proportionate hazards method [14] were used for associations in survival analyses. Maximally selected rank statistics calculations were used to identify the time-to-treatment cut-off for survival [15]. Limited and incongruous previous research meant that a priori power calculations were not feasible [16]. Patients who did not receive SNB were excluded from survival analyses because these patients follow clinically different pathways and the inclusion of these patients may introduce bias.

## Results

1625 patients’ data were identified and included in the analysis, with 1510 (92.9%) who underwent SNB and 115 (7.1%) who did not. For 1170 patients (72.0%) early surgery was planned (≤90 days) and 455 (28.0%) late surgery (>90 days). These subgroups were well matched for patient demographic and tumour characteristics for the entire cohort and post-2016 cohort (Table 1 and S1). Patients undergoing late surgery experienced a slower median report time from the primary excision date (12 days vs 9 days; *p* < 0.001) and were more likely referred from a peripheral hospital in the catchment area (303 (66.6%) vs 570 (48.7%); *p* < 0.001).

The median wait time for surgery was 68 days (IQR = 51-95) from the date of diagnosis and 81 days (IQR = 61–109) from the date of primary excision to SNB. For the early group the median time between diagnosis to SNB was 57 (IQR = 46–71) days and for the late group 117 (IQR = 102–153) days (*p* = <0.001). The median reporting interval was 10 days (IQR = 6–14). The proportion of patients receiving surgery exceeding 90 days in the post-2016 cohort significantly increased compared to pre-2017 (346 (76.0%) vs 109 (23.7%); *p* < 0.0001).

### Disease progression on waiting list

SNB was cancelled for 115 patients initially listed for the procedure of which 11 (9.6%) were for melanoma progression. Table S4 summarises the reasons why patients who were scheduled for SNB did not receive the surgery. Those who did not undergo SNB had waited longer for their procedure (median 80 (IQR = 60-110) days vs 68 (IQR = 50-94) days; *p* < 0.001). Patients who waited beyond 90 days for their surgery had significantly increased risk of clinical disease progression compared to those treated within 90 days (7 (1.5%) versus 4 (0.3%) patients; OR = 4.56 (95% CI = 1.33-15.6); *p* = 0.008). Time-to-treatment was identified as a significant independent predictor for melanoma progression before SNB by multivariable analysis adjusted for age, Breslow thickness, sex and ulceration (Fig. S1: adjusted OR (aOR)=4.31 (95% CI 1.27-16.77); *p* = 0.022, Fig. S2).

### Micrometastatic tumour burden

SNB positivity rate was 19.2% (290/1510 patients). The rates of SNB positivity, ECS and the proportions of AJCC stage N1a and N2a disease did not significantly vary between the early and late subgroups (Table 2). The median maximum micrometastatic deposit diameter was significantly greater in the late subgroup (2.95 mm versus 1.40 mm; p = 0.003). These patients were also more likely to have high-risk deposits, defined as >1 mm (OR = 1.81 (95% CI = 1.06-3.11); p = 0.030). These differences were consistent after multivariable analysis adjusted for age, Breslow thickness, sex and ulceration for median deposit size (Fig. S3: aOR=1.10 (95% CI = 1.04–1.16); *p* = <0.001) and for high-risk deposits (Fig. S4: aOR=2.04 (95% CI = 1.16-3.66); *p* = 0.014).

**Table 2:** Cross-table comparing the phenotypic characteristics of micrometastatic tumour burden of the sentinel nodes for patients with an early (>90 days) or late (≤90 days) SNB (n = 1510).

	*N*	Timing of SNB, *n* (%)				Test statistic	
		Early (≤90 days) (*N* = 1103)		Late (>90 days) (*N* = 407)			*p*-value
**Nodal status: Positive**	1510	205	(18.6)	85	(20.9)	X^2^_1_ = 1.01	0.314^a^
**Deposit size (mm), median (IQR)**	287	1.40	(0.47-4.05)	2.95	(0.78-8.00)	F_1,285_ = 9.32	<0.001^b^
**Extracapsular spread: Present**	287	21	(10.3)	12	(14.2)	X^2^_1_ = 0.91	0.341^a^
**Nodal stage**	1625					X^2^_3_ = 2.10	0.551^a^
N0		898	81.4)	322	(79.1)		
N1		141	(12.8)	57	(14.0)		
N2		58	(5.3)	27	(6.6)		
N3		6	(0.5)	1	(0.2)		
**Deposit size : High Risk (>1 mm)**	287	112	(55.2)	58	(69.0)	X^2^_1_ = 4.74	0.030^a^

N; number of non-missing values; SNB, sentinel node biopsy; IQR, interquartile range.

^a^Pearson.

^b^Kruskal-Wallis.

### Survival

The median follow-up was 75 months (IQR = 35-115 months). In the full cohort, there were 309 (20.5%) recurrences diagnosed during follow-up (90 local, 66 regional and 153 distant as first sites of recurrence). A total of 229 distant metastasis occurred and 159 melanoma-related deaths were recorded. Median follow-up was 39 months (IQR = 19–59 months) for the post-2016 cohort, and there were 94 recurrences detected (28 local, 25 regional and 41 distant as first sites of recurrence). In total, 60 distant recurrences, and 29 melanoma-related deaths were recorded. Multivariable analyses identified age at diagnosis, primary AJCC stage and ulceration status as independent predictors of DSS for both the full and post-2016 cohorts. DSS significantly improved for patients treated after 2016 in comparison to those up to 2016 (HR = 0.56 (95% CI = 0.37–0.84); *p* = 0.006). High-risk micrometastatic SNB deposits were significantly associated with a worse DSS for the full cohort (HR = 1.93 (95% CI = 1.16–3.20); *p* = 0.011) and also for those treated post-2016 (HR = 4.96 (95% CI = 1.15–21.50); *p* = 0.032; log-rank *p* = 0.017). Similar outcomes were seen for RFS and DMFS (data not shown).

**Fig. 1:**
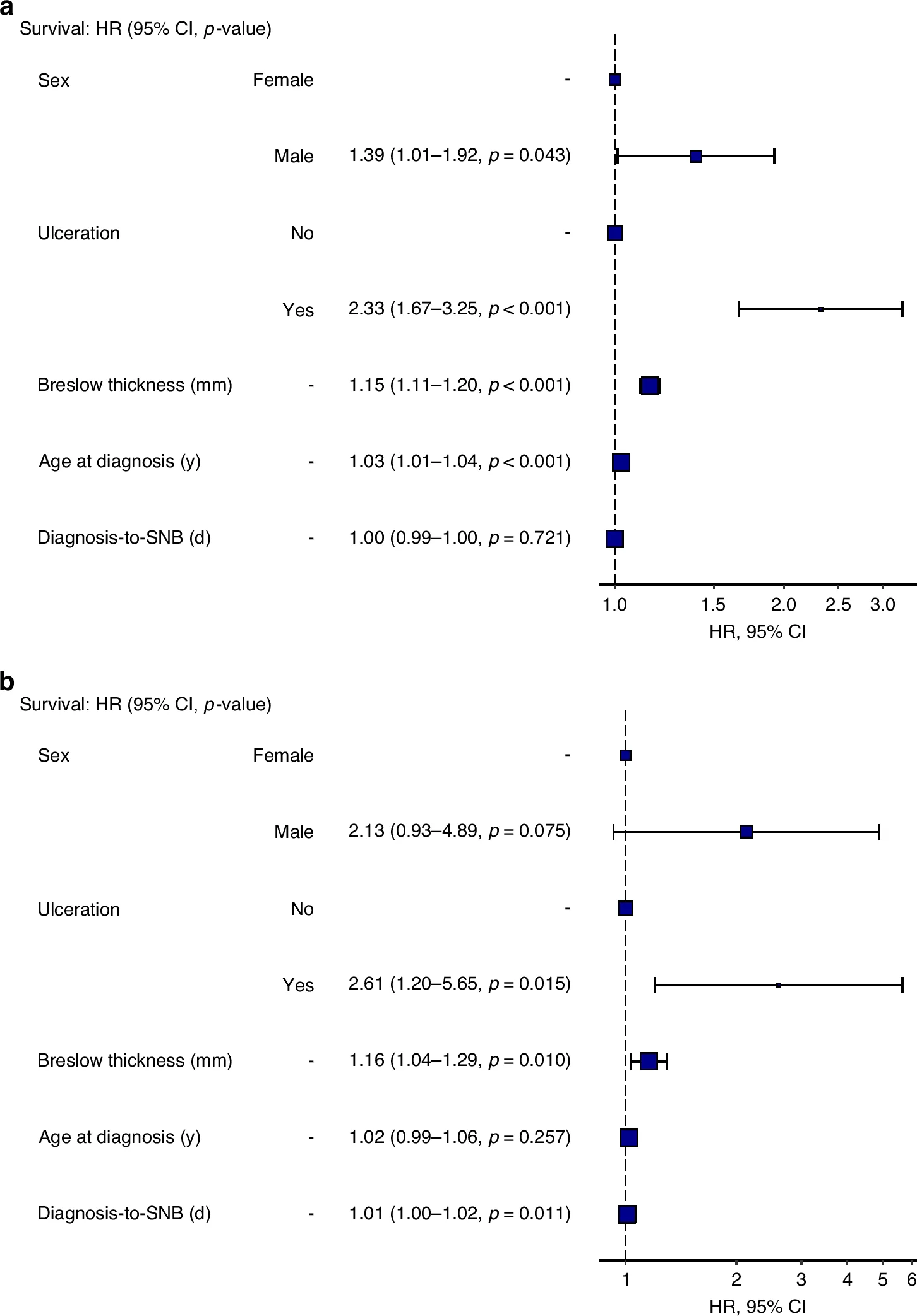
Cox’s regression multivariable analysis of disease-specific survival. **a** Hazard ratio plot for the full cohort adjusted for sex, ulceration, Breslow thickness and age with diagnosis-to-SNB as a continuous variable (n = 1510). **b** Hazard ratio plot for the post-2016 cohort adjusted for the same clinical variables (n = 730).

**Fig. 2:**
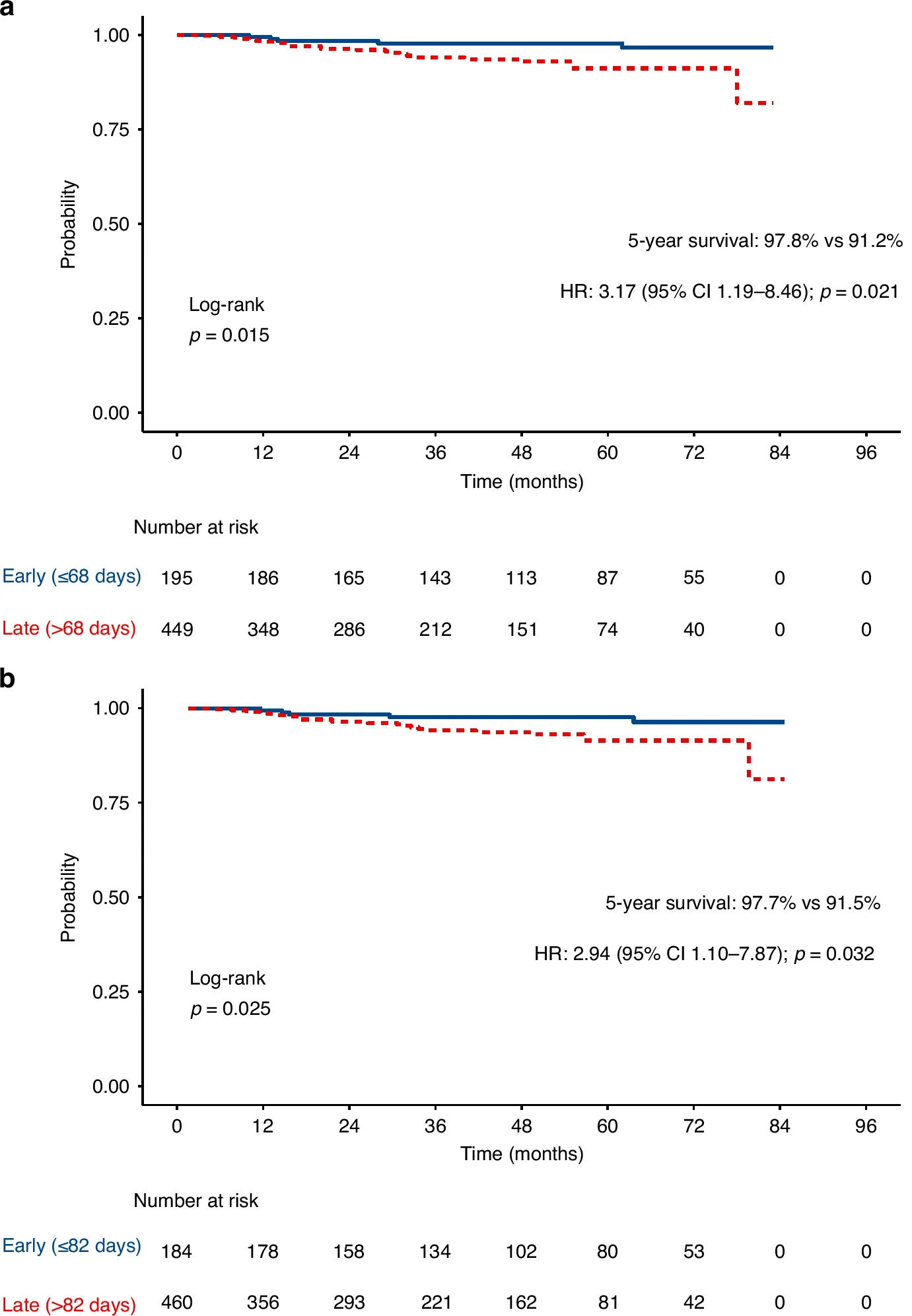
Kaplan-Meier survival curves stratified by time to SNB (post-2016 cohort). **a** Disease-specific survival stratified by a 68-day threshold from the time of diagnosis to SNB (n = 730). **b** Disease-specific survival stratified by an 82-day threshold from the time of excision of the primary melanoma to SNB (n = 730).

**Fig. 3:**
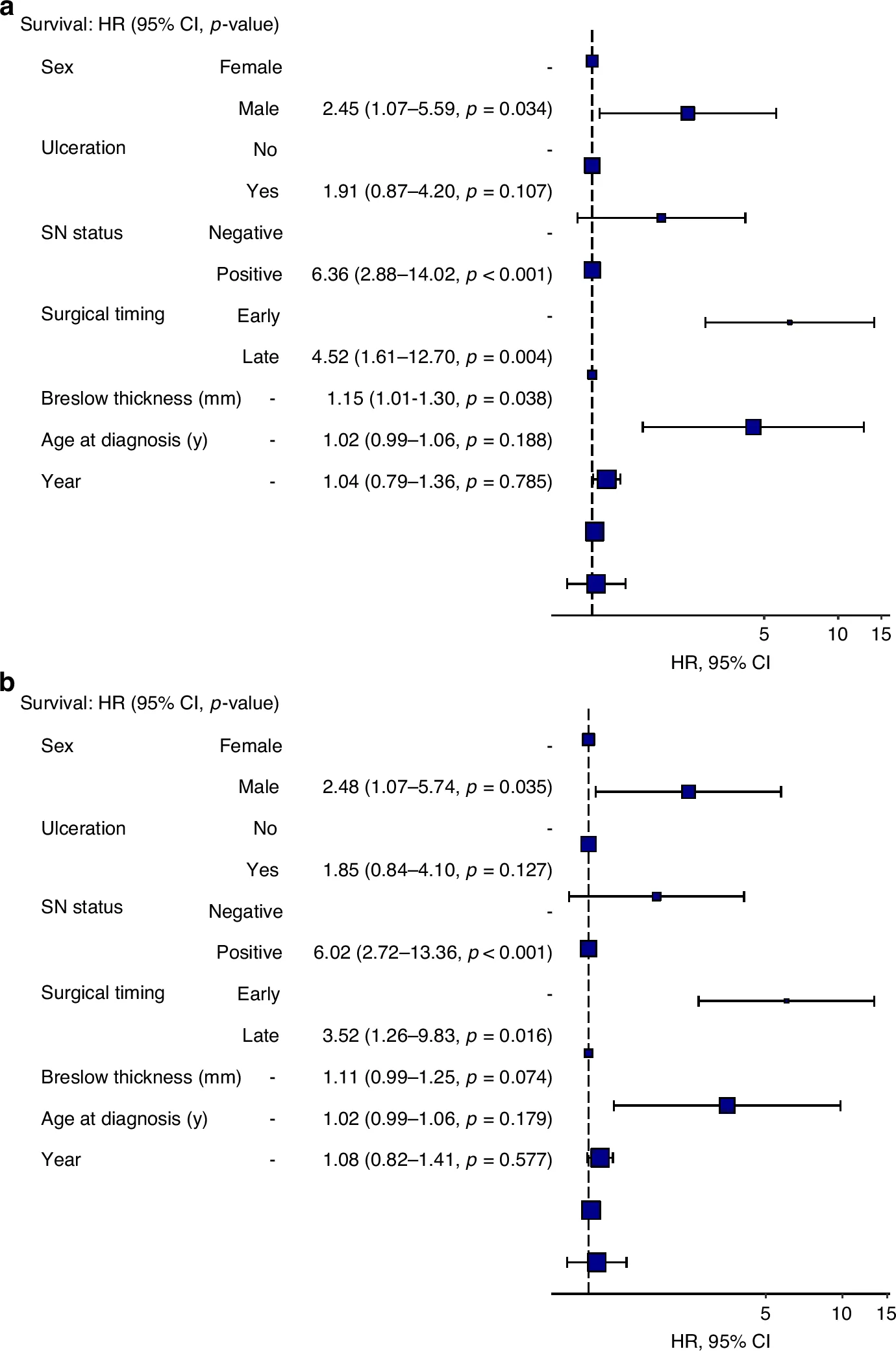
Cox’s regression multivariable analysis of disease-specific survival stratified by time thresholds (post-2016 cohort). **a** Hazard ratio plot adjusted for sex, ulceration, sentinel node status, age at diagnosis, Breslow thickness and year with diagnosis-to-SNB (68 days) (n = 730). **b** Hazard ratio plot adjusted for the same clinical variables with primary excision-to-SNB (82 days) (n = 730).

For the full and post-2016 cohorts, univariable and multivariable analyses adjusted for sex, ulceration, Breslow thickness and age for DSS, RFS and DMFS did not highlight time-to-SNB as an independent predictor when assessed categorically (≤90 versus >90 days). However, multivariable analysis, using both diagnosis-to-SNB and primary excision-to-SNB as continuous variables, found that increasing time was associated with a significantly poorer DSS for the post-2016 cohort (Fig. 1: aHR=1.01 (95% CI = 1.00–1.02); p = 0.011 and aHR=1.01 (95% CI = 1.00–1.02); *p* = 0.016 respectively). A sensitivity analysis using the maximally selected rank statistics method for the post-2016 cohort, identified a significant cut-off of 68-days for diagnosis-to-SNB and 82-days for primary excision-to-SNB (Fig. 2). The interval between the two was consistent with a median reporting interval of 12 days (IQR = 8-17 days) post-2016. Kaplan-Meier curves stratified by >68 and ≤68 days for diagnosis-to-SNB showed a significantly worse 5-year disease-specific survival, 97.8% versus 91.2%, in those treated after 2016 (Fig. 2: HR = 3.17 (95% CI = 1.19–8.46); *p* = 0.021), with similar results for excision-to-SNB (97.7% versus 91.5%; HR = 2.94 (95% CI = 1.10–7.87); *p* = 0.032)). These subgroups were well matched for patient demographic and tumour characteristics (Table S2, S3). Multivariable analyses adjusted for sex, ulceration, Breslow thickness indicated that a late SNB (>68 days) was associated with a significantly greater risk of melanoma-related death in the post-2016 cohort (Fig. 3: aHR=4.52 (95% CI = 1.61–12.70); *p* = 0.004). Whilst the 68-day cut-off was not associated with pre-treatment progression, patients with positive sentinel nodes were more likely to have a larger micrometastatic deposits (median 2.30 mm (IQR = 0.55-7.00 mm) versus 1.15 mm (IQR = 0.48-4.00 mm) respectively; p < 0.009) and high-risk deposits >1 mm (OR = 1.96 (95% CI = 1.21–3.18); *p* = 0.006), which was consistent after adjusting for age, Breslow thickness, sex and ulceration (Fig. S5: aOR=1.09 (95% CI = 1.09–1.15); *p* = 0.002 for deposit size, and Fig. S6: aOR=2.39 (95% CI = 1.43–4.05); *p* = 0.001 for high-risk deposits).

## Discussion

The data confirms that our service began to struggle to achieve a waiting time of 90 days or less for SNB from 2017 onwards (96% in 2010 to 12% in 2023). Increasing disease prevalence, service funding constraints and logistical issues with tracer agents have all contributed, with the covid-19 Pandemic adding further burden. Similar trends across the UK have been anecdotally reported. Time-to-treatment has not been previously investigated using a comparably thorough approach across three critical time points in the melanoma treatment pathway. In the context of a heterogenous and insufficient evidence base, with few patients experiencing delays exceeding 90 days, this one of the first studies to address these important health concerns in the modern melanoma era.

Our analysis shows that delays translate into potential patient harm, not only in the admittedly small, but statistically significant, increased risk of disease progression on the waiting list, but also in the increased micrometastatic burden for SNB-positive patients, and disease-specific survival for patients treated in the adjuvant systemic therapy era. We attribute this significantly worse 5-year absolute survival difference (delta=6.6%; HR = 3.15; *p* = 0.015) to the increased risk of distant disease spread from the greater micrometastatic burden seen in the sentinel nodes. This is concerning given the availability of effective systemic therapy options [5, 9]. Our data confirms that there is a time-critical element to SNB in managing patients with occult nodal micrometastases, with potential harm for those who experience delays.

Our analysis demonstrated that the risk of progression and palpable nodal disease while on the waiting list increases after 90 days. Nodal progression is also an indication for AST, according to current UK guidelines, and more recently neoadjuvant therapy [5]. Most of these patients will undergo more extensive surgery from therapeutic lymph node dissection, with the potential for negative quality of life impact on the patient with perioperative complications and long-term lymphoedema. Thus, granted the increased rate of progression is small (0.68%), it is still nonetheless clinically relevant. Data from the MSLT-1 study [6] suggested that patients with micrometastases in sentinel nodes will develop palpable disease with a greater metastatic burden if untreated, indicating the temporal nature of this issue. Although MSLT-1 measured time in months, our significant results suggest that, for a minority, 90 days is enough for progression.

The pivotal finding of the analysis is the temporal association between SNB and micrometastatic disease burden, which corroborates previous data [17, 18]. SNB performed after 90 days led to significantly larger tumour deposits (median 2.95 mm vs 1.40 mm; *p* < 0.001), and a higher proportion of high-risk deposits >1 mm (55.2% vs 69.0%; OR = 1.81; *p* = 0.030). Our data highlights the concerning speed of progression, measured in days rather than months. The current AJCC (8^th^ Edition) classification for melanoma, indicated that increasing deposit size is significantly associated with worse melanoma-specific survival [2]. In the DeCOG-SLT study, high risk deposits (>1 mm) acted as independent predictors of a poorer DMFS [19]. A recent multicentre study showed that patients with AJCC pT1b-pT2a primaries and low micrometastatic burden (<0.3 mm) have DSS outcomes identical to those with negative SNBs, suggesting a therapeutic benefit for SNB also observed in MSLT-1 and AJCC data [8]. Other studies confirm that, while low-risk micrometastases <0.3 mm are less common in higher-risk primaries, resecting them at SNB may still offer benefit [2, 8]. These findings support the importance of timely SNB to avoid missing a therapeutic window for a clinically significant subset of patients.

Whereas the earlier endpoints (progression on waiting list and micrometastatic burden) applied to the full cohort, the survival analysis focused on the modern treatment era to assess the effect of delay under current care standards. Whilst the treatment paradigm for palpable disease continues to evolve [20, 21], the indications for offering AST for micrometastatic disease have remained consistent since its introduction in the national guidelines [5, 9]. Multivariable analysis confirmed sex, age, primary AJCC tumour stage (Breslow thickness) as independent survival predictors, aligning with AJCC classifications and prior evidence [2]. Whilst our initial univariable and multivariable analyses stratified by the 90-day cut-point failed to indicate it as an independent predictor of survival outcome, multivariable analysis showed that time-to-SNB was a significant independent predictor of DSS outcome when assessed as a continuous variable, suggesting that the arbitrary cut point of 90 days was incorrect. This conclusion was confirmed by the subsequent sensitivity analysis, which highlighted the 68-day cut point, with significant separation of the disease-specific survival curves (HR = 3.17; p = 0.015). In the post-2016 cohort, longer waiting times were significantly associated with increased melanoma-specific mortality. Existing literature includes very few patients treated after 2016 and none beyond 2018, limiting its relevance to the post-pandemic treatment era. A high-risk micrometastatic burden greater than 1 mm was also associated with increased mortality, indicating that time-to-SNB, sentinel node burden, and DSS are closely interconnected.

We would also highlight that the sensitivity analysis has allowed for a specific definition of treatment delay (and, indeed, harm), based on relevant clinical endpoints. Our data suggest that the current 90-day target should be shortened to 68 days from diagnosis or 82 days from excision biopsy, assuming similar median reporting intervals. The latter is less dependent on variable pathology reporting times and may therefore serve as a more consistent performance measure, whilst acknowledging that prolonged pathological delays may further extend treatment intervals.

As a cautionary note, Tejera Vaquerizo et al. in 2015 [22] and 2017 [23] paradoxically noticed that melanomas which received a timely SNB (within ≤40 days) suffered a worse DSS compared to their counterparts at >40 days (HR = 2.06 (95% CI = 1.1–3.53); *p* = 0.08), which they attributed to the disproportionate prioritisation of primary melanomas with high-risk characteristics. Interestingly, Mandala et al. [24] also observed a 30% lower risk of recurrence and/or death was experienced in negative-SNBs with surgery performed ≥32 days after primary excision (HR = 0.70 (95% CI = 0.63–0.79); *p* < 0.001 for RFS and 0.69 (95% CI = 0.61–0.78); *p* < 0.001 for OS), but not SNB positive cases.

The results of this retrospective study at our supra-regional centre should be interpreted with caution without further external validation. The median follow-up in the post-2016 cohort, 39 months, is relatively short. Potential confounders like socioeconomic data, patient performance status, molecular subtyping and the incidence of systemic therapy were incomplete.

## Conclusion

This analysis suggests that delaying SNB beyond 90 days from diagnosis is significantly associated with adverse clinical outcomes, including disease progression and increased metastatic burden. Our findings indicate that sentinel node metastases can rapidly evolve into high-risk deposits, reinforcing the need for prompt SNB. This is the first study to define a threshold for harm: 68 days from diagnosis or 82 days from biopsy. Delays beyond this point were linked to worse disease-specific survival, despite available systemic therapies. Urgent validation is needed, and national guidelines should be updated accordingly if confirmed.

## Supplementary information


Supplementary Material


## Data Availability

Patient data used to support the findings of the study is available from the corresponding author upon reasonable request.

## References

[1] Melanoma Skin Cancer Statistics: Cancer Research UK. 2024. https://www.cancerresearchuk.org/health-professional/cancer-statistics/statistics-by-cancer-type/melanoma-skin-cancer#heading-Zero.

[2] Gershenwald JE, Scolyer RA, Hess KR, Sondak VK, Long GV, Ross MI, et al. Melanoma staging: evidence-based changes in the American joint committee on cancer eighth edition cancer staging manual. CA: A Cancer J Clin. 2017;67:472–92. 10.3322/caac.21409 PMC5978683 29028110

[3] Resource for Management Options of Melanoma During COVID-19. Society of Surgical Oncology; 2020.

[4] Short term recommendations for cutaneous melanoma management during COVID-19 pandemic. National Comprehensive Cancer Network; 2020.

[5] Melanoma: Assessment and Management: National Institute for Health and Care Excellence; 2015 [updated 27 July 2022; cited 31 May 2024]. Available from: https://www.nice.org.uk/guidance/ng14. 36657006

[6] Morton DL, Thompson JF, Cochran AJ, Mozzillo N, Nieweg OE, Roses DF, et al. Final trial report of sentinel-node biopsy versus nodal observation in melanoma. N Engl J Med. 2014;370:599–609. 24521106 10.1056/NEJMoa1310460 PMC4058881

[7] Moncrieff MD, Lo SN, Scolyer RA, Heaton MJ, Nobes JP, Snelling AP, et al. Clinical outcomes and risk stratification of early-stage melanoma micrometastases from an international multicenter study: implications for the management of american joint committee on cancer IIIA disease. J Clin Oncol. 2022;40:3940–51. 35849790 10.1200/JCO.21.02488

[8] Hussain Z, Heaton MJ, Snelling AP, Nobes JP, Gray G, Garioch JJ, et al. Risk stratification of sentinel node metastasis disease burden and phenotype in stage III melanoma patients. Ann Surgical Oncol. 2023;30:1808–19. 10.1245/s10434-022-12804-6 PMC9908720 36445500

[9] Swetter SM, Thompson JA, Albertini MR, Barker CA, Baumgartner J, Boland G, et al. NCCN Guidelines® Insights: Melanoma: Cutaneous, Version 2.2021. J Natl Compr Cancer Netw. 2021;19:364–76. 10.6004/jnccn.2021.0018 33845460

[10] Van Der Ploeg APT, Van Akkooi ACJ, Haydu LE, Scolyer RA, Murali R, Verhoef C, et al. The prognostic significance of sentinel node tumour burden in melanoma patients: an international, multicenter study of 1539 sentinel node-positive melanoma patients. Eur J Cancer. 2014;50:111–20. 24074765 10.1016/j.ejca.2013.08.023

[11] jamovi. The jamovi project. Version 2.5. Sydney, Australia; 2024. Available from: https://www.jamovi.org.

[12] R Core Team R: A Language and Environment for Statistical Computing. Vienna, Austria: R Foundation for Statistical Computing; 2023. pp. 01–09. https://www.R-project.org/Version 4.3Available from(R packages retrieved from CRAN snapshot 2024.

[13] Kaplan EL, Meier P. Nonparametric estimation from incomplete observations. J Am Stat Assoc. 1958;53:457–81.

[14] Cox DR. Regression models and life-tables. J R Stat Soc: Ser B (Methodol). 1972;34:187–202.

[15] Lausen B, Schumacher M. Maximally Selected Rank Statistics. Biometrics. 1992;48:73–85.

[16] Oude CM, van Akkooi ACJ, Rutkowski P, Voit CA, Stepniak J, Erler NS, et al. Effects of time interval between primary melanoma excision and sentinel node biopsy on positivity rate and survival. Eur J Cancer. 2016;67:164–173. 27669503 10.1016/j.ejca.2016.08.014

[17] El Sharouni MA, Scolyer RA, Van Gils CH, Ch’Ng S, Nieweg OE, Pennington TE, et al. Effect of the time interval between melanoma diagnosis and sentinel node biopsy on the size of metastatic tumour deposits in node-positive patients. Eur J Cancer. 2022;167:133–41. 35216870 10.1016/j.ejca.2021.12.036

[18] El Sharouni MA, Scolyer RA, van Gils CH, Ch’ng S, Nieweg OE, Pennington TE, et al. Time interval between diagnostic excision-biopsy of a primary melanoma and sentinel node biopsy: effects on the sentinel node positivity rate and survival outcomes. Eur J Cancer. 2022;167:123–32. 35256245 10.1016/j.ejca.2021.12.035

[19] Leiter U, Stadler R, Mauch C, Hohenberger W, Brockmeyer NH, Berking C, et al. Final Analysis of DeCOG-SLT trial: no survival benefit for complete lymph node dissection in patients with melanoma with positive sentinel node. J Clin Oncol. 2019;37:3000–8. 31557067 10.1200/JCO.18.02306

[20] Patel SP, Othus M, Chen Y, Wright GP, Yost KJ, Hyngstrom JR, et al. Neoadjuvant–adjuvant or adjuvant-only pembrolizumab in advanced melanoma. N Engl J Med. 2023;388:813–23. 36856617 10.1056/NEJMoa2211437 PMC10410527

[21] Blank CU, Lucas MW, Scolyer RA, Van De Wiel BA, Menzies AM, Lopez-Yurda M, et al. Neoadjuvant nivolumab and ipilimumab in resectable stage III melanoma. N Engl J Med. 2024;391:1696–708. 38828984 10.1056/NEJMoa2402604

[22] Tejera-Vaquerizo A, Nagore E, Puig S, Robert C, Saiag P, Martin-Cuevas P, et al. Effect of time to sentinel-node biopsy on the prognosis of cutaneous melanoma. Eur J Cancer. 2015;51:1780–93. 26072362 10.1016/j.ejca.2015.05.023 PMC4768477

[23] Tejera-Vaquerizo A, Descalzo-Gallego MA, Traves V, Requena C, Bolumar I, Pla A, et al. The intriguing effect of delay time to sentinel lymph node biopsy on survival: a propensity score matching study on a cohort of melanoma patients. Eur J Dermatol. 2017;27:487–95. 28944755 10.1684/ejd.2017.3065

[24] Mandalà M, Galli F, Patuzzo R, Maurichi A, Mocellin S, Rossi CR, et al. Timing of sentinel node biopsy independently predicts disease-free and overall survival in clinical stage i-ii melanoma patients: a multicentre study of the Italian melanoma intergroup (IMI). Eur J Cancer. 2020;137:30–9. 32739767 10.1016/j.ejca.2020.07.001 PMC7391020

