# Supplementary material for: Implications of wait times for sentinel node biopsy on melanoma disease progression, micrometastatic tumour burden and survival outcomes in the modern treatment era
Source: Br J Cancer. 2026 Jun 16;135(6):909–16. doi: 10.1038/s41416-026-03497-9 (PMC13534719; doi:10.1038/s41416-026-03497-9)
Supplement: Supplementary file 1 [file 41416_2026_3497_MOESM1_ESM.docx]

**Supplementary Data**

**Table S1:** Cross-table comparing patient demographic and primary tumour characteristics for patients at the time of diagnosis with an early (>90 days) or late (≤90 days) SNB for the post-2016 cohort (n=730)

| **Variable** | **N** | **Early SNB (≤90 days)**  **(N=384)** | | **Late SNB (>90 days)**  **(N=346)** | | **Test statistic** | |
| --- | --- | --- | --- | --- | --- | --- | --- |
|  |  |  |  |  |  |  | P-value |
| **Age (yrs), median (IQR)** | 730 | 63 | (53-71) | 65 | (55-72) | F_1,728_=1.15 | 0.284^1^ |
| **Breslow thickness (mm), median (IQR)** | 729 | 1.9 | (1.3-3.0) | 2.1 | (1.2-3.6) | F_1,727_=1.07 | 0.300^1^ |
| **Reporting interval (days), median (IQR)** | 730 | 13.0 | (8.0-17.6) | 12.0 | (9.0-17.0) | F_1,728_=0.33 | 0.567^1^ |
| **Sex : Male** | 730 | 212 | (55.2) | 178 | (51.4) | X^2^_1_=1.04 | 0.309^2^ |
| **Referral location : Peripheral hospital** | 730 | 163 | (42.4) | 223 | (64.5) | **X^2^_1_=35.36** | **<0.001^2^** |
| **Site**  Head and neck  Lower extremity  Torso  Upper extremity | 730 | 60  86  155  83 | (15.6)  (22.4)  (40.4)  (21.6) | 48  86  134  78 | (13.9)  (24.9)  (38.7)  (22.5) | X^2^_3_=1.04 | 0.792^2^ |
| **Subtype**  Superficial spreading  Nodular  Lentigo  Acral lentiginous  Other | 730 | 274  63  15  9  23 | (71.4)  (16.4)  (3.9)  (2.3)  (6.0) | 245  65  12  3  21 | (70.8)  (18.8)  (3.5)  (0.9)  (6.1) | X^2^_4_=3.11 | 0.540^2^ |
| **Lymphovascular invasion : Yes** | 721 | 27 | (7.1) | 14 | (4.1) | X^2^_1_=3.08 | 0.079^2^ |
| **Ulceration : Yes** | 728 | 111 | (29.0) | 93 | (27.0) | X^2^_1_=0.37 | 0.544^2^ |
| **T-stage^2^**  IA  IB  IIA  IIB  IIC | 728 | 7  177  95  74  30 | (1.8)  (46.2)  (24.8)  (19.3)  (7.8) | 3  150  93  59  40 | (0.9)  (43.5)  (27.0)  (17.1)  (11.6) | X^2^_4_=5.00 | 0.287^2^ |
| **SNB Performed : Yes** | 730 | 341 | (88.8) | 303 | (87.6) | X^2^_1_=0.26 | 0.607^2^ |
| Values are n (%) unless otherwise indicated; N; number of non-missing values; SNB, sentinel lymph node biopsy; IQR, interquartile range.  1 Kruskal-Wallis 2 Pearson  2 American Joint Committee on Cancer (AJCC) Classification | | | | | | | |

**Table S2:** Cross-table comparing patient demographic and primary tumour characteristics for patients at the time of diagnosis with an early (>68 days) or late (≤68 days) SNB for the entire cohort (n=1625)

| **Variable** | **N** | **Early SNB (≤68 days)**  **(N=824)** | | **Late SNB (>68 days)**  **(N=801)** | | **Test statistic** | |
| --- | --- | --- | --- | --- | --- | --- | --- |
|  |  |  |  |  |  |  | P-value |
| **Age (yrs), median (IQR)** | 1625 | 63 | (52-71) | 65 | (54-72) | F_1,1623_=3.58 | 0.059^1^ |
| **Breslow thickness (mm), median (IQR)** | 1622 | 1.8 | (1.2-2.9) | 1.8 | (1.2-3.2) | F_1,1620_=1.82 | 0.178^1^ |
| **Reporting interval (days), median (IQR)** | 1625 | 8.0 | (5.4-14.0) | 11.0 | (7.0-15.0) | **F_1,1623_=45.20** | **<0.001^1^** |
| **Sex : Male** | 1625 | 443 | (53.8) | 400 | (49.9) | X^2^_1_=2.38 | 0.123^2^ |
| **Referral location : Peripheral hospital** | 1625 | 358 | (43.4) | 515 | (64.3) | **X^2^_1_=71.01** | **<0.001^2^** |
| **Year : Post-2016** | 1625 | 222 | (26.9) | 508 | (63.4) | **X^2^_1_=218.45** | **<0.001** |
| **Site**  Head and neck  Lower extremity  Torso  Upper extremity | 1625 | 119  201  330  174 | (14.4)  (24.4)  (40.0)  (21.1) | 127  202  299  173 | (15.9)  (25.2)  (37.3)  (21.6) | X^2^_3_=1.47 | 0.690^2^ |
| **Subtype**  Superficial spreading  Nodular  Lentigo  Acral lentiginous  Other | 1625 | 581  143  41  22  37 | (70.5)  (17.4)  (5.0)  (2.7)  (4.5) | 562  149  31  9  50 | (70.2)  (18.6)  (3.9)  (1.1)  (6.2) | X^2^_4_=8.90 | 0.064^2^ |
| **Lymphovascular invasion : Yes** | 1598 | 61 | (7.5) | 36 | (4.6) | **X^2^_1_=6.02** | **0.014^2^** |
| **Ulceration : Yes** | 1621 | 210 | (25.5) | 209 | (26.2) | X^2^_1_=0.10 | 0.757^2^ |
| **T-stage^2^**  IA  IB  IIA  IIB  IIC | 1620 | 22  416  186  131  67 | (2.7)  (50.6)  (22.6)  (15.9)  (8.2) | 23  372  194  128  81 | (2.9)  (46.6)  (24.3)  (16.0)  (10.2) | X^2^_4_=3.65 | 0.455^2^ |
| **SNB Performed : Yes** | 1625 | 781 | (94.8) | 729 | (91.0) | **X^2^_1_=8.78** | **0.003^2^** |
| Values are n (%) unless otherwise indicated; N; number of non-missing values; SNB, sentinel lymph node biopsy; IQR, interquartile range.  1 Kruskal-Wallis 2 Pearson  2 American Joint Committee on Cancer (AJCC) Classification | | | | | | | |

**Table S3:** Cross-table comparing patient demographic and primary tumour characteristics for patients at the time of diagnosis with an early (>68 days) or late (≤68 days) SNB for the post-2016 cohort (n=730)

| **Variable** | **N** | **Early SNB (≤68 days)**  **(N=222)** | | **Late SNB (>68 days)**  **(N=508)** | | **Test statistic** | |
| --- | --- | --- | --- | --- | --- | --- | --- |
|  |  |  |  |  |  |  | P-value |
| **Age (yrs), median (IQR)** | 730 | 62.5 | (51.9-71.0) | 64 | (54.0-72.0) | F_1,728_=1.52 | 0.218^1^ |
| **Breslow thickness (mm), median (IQR)** | 729 | 1.9 | (1.3-2.8) | 2.1 | (1.2-3.5) | F_1,727_=1.22 | 0.269^1^ |
| **Reporting interval (days), median (IQR)** | 730 | 12.0 | (8.0-17.1) | 13.0 | (9.0-17.0) | F_1,728_=1.12 | 0.289^1^ |
| **Sex : Male** | 730 | 131 | (59.0) | 259 | (51.0) | **X^2^_1_=4.00** | **0.046^2^** |
| **Referral location : Peripheral hospital** | 730 | 71 | (32.0) | 315 | (62.0) | **X^2^_1_=55.90** | **<0.001^2^** |
| **Site**  Head and neck  Lower extremity  Torso  Upper extremity | 730 | 36  44  92  50 | (16.2)  (19.8)  (41.4)  (22.5) | 72  128  197  111 | (14.2)  (25.2)  (38.8)  (21.9) | X^2^_3_=2.64 | 0.451^2^ |
| **Subtype**  Superficial spreading  Nodular  Lentigo  Acral lentiginous  Other | 730 | 152  39  12  7  12 | (68.5)  (17.6)  (5.4)  (3.2)  (5.4) | 367  89  15  5  32 | (72.2)  (17.5)  (3.0)  (1.0)  (6.3) | X^2^_4_=7.45 | 0.114^2^ |
| **Lymphovascular invasion : Yes** | 721 | 17 | (7.8) | 24 | (4.8) | X^2^_1_=2.60 | 0.107^2^ |
| **Ulceration : Yes** | 728 | 64 | (28.8) | 140 | (27.7) | X^2^_1_=0.10 | 0.748^2^ |
| **T-stage**  IA  IB  IIA  IIB  IIC | 728 | 2  107  58  40  15 | (0.9)  (48.2)  (26.1)  (18.0)  (6.8) | 8  220  130  93  55 | (1.6)  (43.5)  (25.7)  (18.4)  (10.9) | X^2^_4_=4.02 | 0.403^2^ |
| **SNB Performed : Yes** | 730 | 195 | (87.8) | 449 | (88.4) | X^2^_1_=0.04 | 0.833^2^ |
| Values are n (%) unless otherwise indicated; N; number of non-missing values; SNB, sentinel lymph node biopsy; IQR, interquartile range.  1 Kruskal-Wallis 2 Pearson | | | | | | | |

**Table S4:** Table summarising patients who did not receive SNB (n=115)

| **Variable** | **Early SNB (≤90 days)**  **(N=67)** | | **Late SNB (>90 days)**  **(N=48)** | | **Total**  **(N=115)** | |
| --- | --- | --- | --- | --- | --- | --- |
| **Reason for not performing SNB, N (%)**  Technical^1^  Patient factors^2^  Progression^3^  Unknown | 48  15  4  0 | (71.6%)  (22.4%)  (6.0%)  (0.0%) | 29  11  7  1 | (60.4%)  (22.9%)  (14.6%)  (2.1%) | 77  26  11  1 | (60.9%)  (22.6%)  (9.6%)  (0.9%) |
| **Total** | 67 | (58.3%) | 48 | (41.7%) | 115 | (100.0%) |
| SNB, sentinel lymph node biopsy; IQR, interquartile range.  1 Failure of mapping e.g., failure to migrate or too many nodes were identified.  2 Failed attendance to appointment or allergic reaction to tracer agent.  3 Clinical and/or radiological metastatic disease after primary diagnosis and prior to or at the time of lymphoscintigraphy. | | | | | | |

*Figures*

**Figure S1:** Odds ratio plot for early (≤90 days) and late (>90 days) SNB adjusted for age, Breslow thickness, sex, ulceration and progression for the entire cohort (N=1625)

SNB, sentinel lymph node biopsy; OR, odds ratio; 95% CI, 95% confidence interval

**Figure S2:** Odds ratio plot for melanoma progression before or at SNB adjusted for age, Breslow thickness, sex, ulceration and diagnosis-to-SNB (N=1625)

SNB, sentinel lymph node biopsy; OR, odds ratio; 95% CI, 95% confidence interval

**Figure S3:** Odds ratio plot for early (≤90 days) and late (>90 days) SNB adjusted for age, Breslow thickness, SNB deposit size, sex and ulceration (N=286)

SNB, sentinel lymph node biopsy; OR, odds ratio; 95% CI, 95% confidence interval

**Figure S4:** Odds ratio plot for early (≤90 days) and late (>90 days) SNB adjusted for age, Breslow thickness, sex, ulceration and high (>1mm) and low (≤1mm) risk deposit (N=286)

SNB, sentinel lymph node biopsy; OR, odds ratio; 95% CI, 95% confidence interval

**Figure S5:** Odds ratio plot for early (≤68 days) and late (>68 days) SNB adjusted for age at diagnosis, Breslow thickness, SNB deposit size, sex and ulceration (N=286).

SNB, sentinel lymph node biopsy; OR, odds ratio; 95% CI, 95% confidence interval

**Figure S6:** Odds ratio plot for early (≤68 days) and late (>68 days) SNB adjusted for age at diagnosis, Breslow thickness, sex, ulceration and high (>1mm) and low (≤1mm)-risk deposit (N=286).

SNB, sentinel lymph node biopsy; OR, odds ratio; 95% CI, 95% confidence interval
